# GABA_A_ Receptor Subtypes: Novel Targets for Novel Medicines

**DOI:** 10.1155/2012/529861

**Published:** 2012-04-18

**Authors:** Naheed R. Mirza, John Atack, Keith Wafford

**Affiliations:** ^1^Neuro Search A/S, Pederstrupvej 93, 2750 Ballerup, Denmark; ^2^Johnson & Johnson Pharmaceutical Research and Development, 30 Turnhoutseweg, B-2340 Beerse, Belgium; ^3^Eli Lilly UK, Erl Wood Manor, Windlesham, Surrey, GU20 6PH, UK

GABA_A_ receptors are ligand gated ion channels that are targeted by drugs with multiple therapeutic applications. Most notably, such drugs include the benzodiazepines, the hypnotic Z-drugs (zolpidem, zopiclone, and zaleplon), and the barbiturates. The benzodiazepines rapidly achieved pre-eminence as minor tranquilizers when they were introduced in the 1960s but thereafter lost favour, in a story that exemplifies how a “good drug can go bad” [1]. Nevertheless, benzodiazepines remain a mainstay of psychopharmacology but the decline in their public image is encapsulated by the colourful comment of Gorman [2] who stated that “Prescribing benzodiazepines is like watching pornography. If you ask a person at random if he watches pornography, he will vehemently deny it, but someone must be because it is a billion dollar a year business. Similarly, if you ask a physician if he prescribes Valium or Xanax or Ativan, he or she will say of course not…. Yet, like pornography, benzodiazepine prescriptions generate billions of dollars of revenue around the world, so somebody must be prescribing them”. Recently, there has been a resurgence of interest in GABA_A_ receptor pharmacology which has formed the basis for the development of novel modulators of discrete populations of this receptor family [3].

Over the last 25 years or so considerable advances in the molecular biology of GABA_A_ receptors have identified multiple subtypes of GABA_A_ receptor. Hence, GABA_A_ receptors are heteropentameric assemblies of proteins derived from a family comprising 16 members (a_1–6_, *β*_1–3_, *γ*_1–3_, *δ*, *ε*, *θ*, *π*). Despite the enormous number of theoretical pentameric combinations, thankfully only around 20 configurations have been described in native receptors [4]. A variety of approaches have been used to develop a greater understanding of the roles of these different subtypes in the physiology and pathophysiology of the CNS. These strategies include the generation of transgenic mouse models that have been used to delineate the functions of different subtypes, and the identification of subtype-selective pharmacological compounds that have formed the basis of hypotheses that subtype-selectivity can lead to therapeutic selectivity (e.g., anxioselective drugs without sedation). A combination of these strategies has resulted in emerging scientific evidence for novel therapeutic applications for subtype-selective drugs (e.g., in neuropathic pain, autism, schizophrenia, Alzheimer's disease, and stroke), and consistent with these various hypotheses clinical data on subtype-selective drugs in human trials indicate a novel pharmacology relative to known drugs targeting GABA_A_ receptors. In this special issue, the articles focus on various aspects of the molecular genetics as well as preclinical and clinical pharmacology of GABA_A_ receptors.

S. Nickoll's et al. in his paper focus on the novel therapeutic opportunity afforded by drugs that positively modulate GABA_A_-*α*_2_/*α*_3_ receptors selectively as novel analgesics without sedation, cognition impairment, or abuse. Testing such compounds in animal models of inflammatory and neuropathic pain, complemented by in vivo electrophysiology, they highlight the need for sufficient efficacy at relevant subtypes of GABA_A_ receptor in addition to selectivity. C. Vinkers and B. Olivier explore mechanisms underlying benzodiazepine tolerance and conclude that no unifying underlying mechanisms can be discerned but emphasize that since this is an important therapeutic issue with respects to currently marketed GABAergic drugs, it is an important area to investigate with respects to emerging subtype selective compounds.

GABA_A_-*α*_5_ and GABA_A_-*δ* receptors are located extrasynaptically, mediate tonic inhibitory neurotransmission in the CNS, and have different biophysical, physiological, and pharmacological properties relative to GABA_A_-*α*_1_/*α*_2_/*α*_3_ receptors which are predominantly synaptically located. J. Braudeau et al. focus on selective negative modulators of GABA_A_-*α*_5_ receptors as a unique cognition enhancing strategy in Down's syndrome (DS), by demonstrating that the selective compound *α*5IA induces gene expression (c-fos and Arc) and rescues impaired gene expression in the Ts65Dn mouse model of DS. The compound *α*5IA has already shown evidence of cognition enhancement in man. Currently, Roche pharmaceuticals is assessing a compound (RG1662) in Down's syndrome, and by extension the relevance of this line of research in potentially reducing cognitive burden in Alzheimer's disease is clear. A. Clarkson reviews the complex role of GABA_A_ receptors in cerebral ischaemia/stroke, suggesting that in an animal model of stroke, treatment with the *α*_5_-selective negative allosteric modulator L-655708 can enhance functional recovery when given after a delay but not, interestingly, when given at the time of stroke. The assumption is that the attenuation of extrasynaptic GABAergic function can be beneficial by counteracting an increase in tonic inhibition that results from increased GABA concentrations in the peri-infarct region. Consequently, therapeutic strategies based upon a disinhibition of GABA_A_-*α*_5_ or GABA_A_-*δ* receptors may aid functional recovery in vivo.

A. C. Errington et al. suggest negative modulators of GABA_A_-*δ*  receptors as a novel treatment option for absence epilepsy given that enhanced GABAergic function has been demonstrated in animal models of absence epilepsy, and that compounds enhancing GABA_A_ function can engender absence seizures. Certainly this suggestion is contrary to the classical approach of treating seizures by increasing inhibitory neurotransmission, but the authors elegantly demonstrate the relevance of key thalamocortical circuits in generating EEG signatures common to animals and man and the high expression and relevance of a *α*_4_ *β*_2_ *δ* receptor population in this circuit. M. W. Hulin et al. focus on literature implicating GABA_A_ receptors and neurosteroids in mediating actions of alcohol. They describe the neurosteroid dehydroepiandrosterone (DHEA) which decreases alcohol intake in rats and broadly behaves as a negative modulator of GABA_A_ receptors, although genomic effects of this molecule complicate matters. Nonetheless, the authors emphasize the potential role of GABA_A_-*δ*  receptors in mediating the effects of DHEA in reducing alcohol intake; albeit this is a controversial area.

X. Chen et al. use a comparative pharmacology approach in human volunteers to demonstrate that subtype-selective GABA_A_ drugs engender pharmacodynamic effects that only partly overlap with those of benzodiazepines. For example, the GABA_A_-*α*_2_/*α*_3_ selective drug TPA023 induces impairment of saccadic peak velocity but does not induce body sway or impair attention in volunteers. The novel pharmacological profile of subtype selective compounds in man mirrors preclinical data using transgenic mouse models and subtype-selective tools, indicating that molecular neuroscience can be translated to the clinic and guide drug development.

The paper by B. H. Bentzen and M. Grunnet gives some insight into the drug development of new chemical entities, specifically focusing on cardiovascular (CV) safety. Clearly many benzodiazepines were registered for clinical use in a regulatory environment considerably different from today's. Therefore, in developing novel subtype-selective modulators, assumptions cannot be made in terms of safety and toxicology. These authors use anaesthetized animals and isolated hearts to emphasize that the CV effects of benzodiazepines are highly dependent on the conscious state of the animal, with implications for CV assessment of future novel GABAergic molecules.

Given the wide-spread therapeutic success of GABAergic drugs across a variety of indications and an emerging understanding of the pharmacology of subtype-selective drugs preclinically and clinically, GABA_A_ receptors are clearly a highly druggable target class for which preclinical to clinical transition/translation strategies exist—desirable qualities in today's highly complex drug development and regulatory environment. The articles in this special issue give a snap-shot of the diverse range of topics being explored regarding the therapeutic utility of selectively targeting GABA_A_ receptors, providing a ground base of understanding for potentially important drugs of the future.

*Naheed R. Mirza* *John Atack* *Keith Wafford*
